# Utilization of Robot-Assisted Gait Training in Pulmonary Rehabilitation for a Patient with Ambulatory Dysfunction Post-Severe COVID-19 Pneumonia: A Case Report

**DOI:** 10.3390/jcm13206213

**Published:** 2024-10-18

**Authors:** June Sung Lee, Jung Hoon Ahn, Jang Woo Lee, Chang Yoon Baek

**Affiliations:** 1 Department of Rehabilitation Center, National Health Insurance Ilsan Hospital, 100 Ilsan-ro, Ilsandong-gu, Gyeonggi-do, Goyang-si 10444, Republic of Korea; 2 R&D Institute, SYM Healthcare Inc., 3F, 39, Seongsuil-ro 8-gil, Seongdong-gu, Seoul 04794, Republic of Korea; 3 Department of Physical Medicine and Rehabilitation, National Health Insurance Ilsan Hospital, Goyang-si 10444, Republic of Korea

**Keywords:** COVID-19, functional ambulation, rehabilitation

## Abstract

Background: Severe COVID-19 can lead to a decline in pulmonary and physical functions simultaneously. Patients experiencing significant ambulatory dysfunction often face restrictions in participating in gait training, resulting in insufficient benefits from cardiopulmonary rehabilitation. This underscores the need for tailored rehabilitation approaches that address their specific conditions. Method: This study presents a case examining the impact of combining pulmonary rehabilitation with robot-assisted gait training (RAGT) on pulmonary and physical functions in a patient with severe COVID-19 pneumonia. A 56-year-old male patient with severe COVID-19 pneumonia exhibited impaired pulmonary function, reduced lower extremity strength, compromised balance, and significant limitations in functional ambulation. He underwent an inpatient pulmonary rehabilitation program combined with RAGT for 5 weeks, participating in 30 min RAGT sessions a total of 22 times. The patient showed improvements in his pulmonary function, lower extremity strength, balance, exercise capacity, and functional mobility, along with a reduction in symptoms such as dyspnea and fatigue. Conclusions: The combination of RAGT with pulmonary rehabilitation is a treatment method that can be applied without complications and has the potential to improve pulmonary and physical functions in patients with ambulatory dysfunction due to COVID-19.

## 1. Introduction

Coronavirus disease 2019 (COVID-19) is still spreading all over the world [1]. Even if the COVID-19 virus is treated, people can still suffer from long COVID-19 syndrome, involving fatigue, loss of smell and taste, arthralgia, exercise intolerance, and cognitive changes [1], which deteriorates physical performance and quality of life [1,2]. The clinical symptoms of COVID-19 remain unclear and vary widely. Most people (80%) experience mild to moderate illness, 15% suffer from severe disease, and 5% experience critical illness, resulting in complications such as pneumonia, pulmonary fibrosis, multi-organ failure, heart problems, acute respiratory distress syndrome, and septic shock [3]. If various complications caused by COVID-19 are accompanied, this can lead to long-term hospitalization and a poor prognosis. Severe COVID-19 pneumonia can lead to prolonged hospitalization and intensive care unit (ICU) treatment [4]. Additionally, it can result in post-intensive care syndrome, which may contribute to a decline in physical function, including walking and balance ability, respiratory capacity, and the overall activities of daily life [5]. Many studies have reported various rehabilitation approaches after COVID-19, which have involved breathing exercises, early mobilization, body positioning management, and aerobic training (treadmill walking, ergometers, and climbing training) [6,7,8]. However, the pulmonary rehabilitation (PR) approach has limitations in patients with a severely restricted functional ambulation capacity due to complications from severe COVID-19 and long-term ICU treatment, especially in cases where a patient is unable to walk or stand. Robot-assisted gait training (RAGT) has been shown to be beneficial for patients with an impaired functional ambulation capacity and the inability to stand or walk independently, including older adults and individuals with various neurological disorders [9,10,11]. By utilizing robotic devices and providing support and guidance, RAGT allows these patients to gain early experience in standing and walking, which may lead to improvements in pulmonary function, muscle strength, and functional ambulation ability [9,10,11,12,13]. Even in outpatient patient following COVID-19 with the ability to walk, robot-assisted training improved the patient’s physical abilities [14]. To date, there has been no study regarding the effect of RAGT on patients with severe functional ambulation limitations after severe COVID-19 pneumonia. We present marked improvements in physical function as well as respiratory symptoms using RAGT in combination with a PR program in a patient with ambulatory dysfunction after a severe COVID-19 infection.

## 2. Case Description

**Figure 1:**
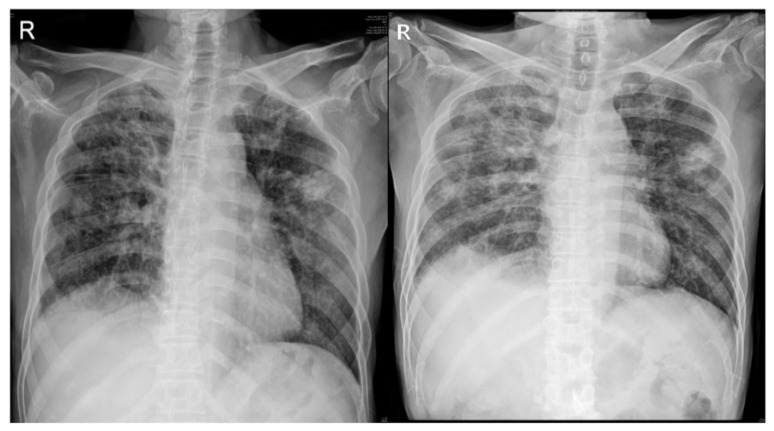
Finding on chest X-ray in the patient with COVID-19. In the first AP and second PA images, it demonstrates diffuse ground-glass opacity and a large nodular lesion in the left upper lobe.

**Figure 2:**
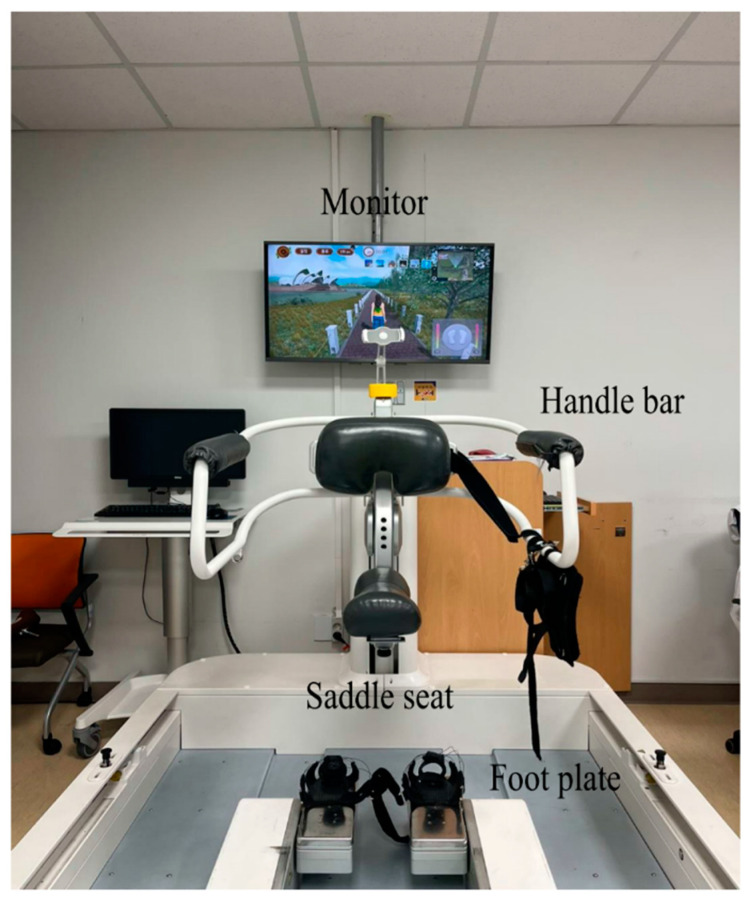
Morning Walk^®^, an end-effector type robot, includes a saddle seat and two handlebars that measure the amount of body weight support, a footplate that reproduces the gait pattern, and a monitor that provides visual feedback on the gait task.

**Figure 3:**
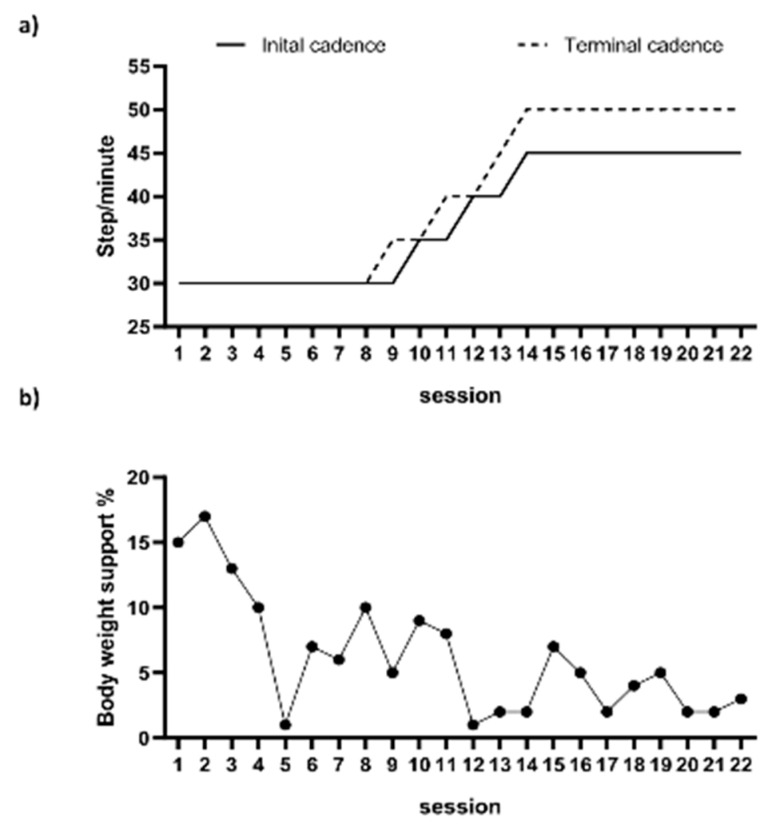
Changes in parameter values between sessions within the robot-assisted gait training program. (**a**) Change in initial and terminal cadence values between sessions. (**b**) Changes in mean degree of BWS between sessions.

A 56-year-old male patient with no significant medical history other than hypertension experienced an acute onset of dyspnea on 18 January 2022. Following a confirmed diagnosis of COVID-19, the patient received inpatient treatment. Despite the administration of Remdesivir and Dexamethasone, hypoxemia did not improve, leading to his admission to the ICU for endotracheal intubation and mechanical ventilatory support. Due to prolonged ventilatory support, a tracheostomy was performed. Subsequently, the patient was transferred to a general ward and later admitted to a nursing hospital on 10 April. On 24 July, the patient was admitted to a university hospital, where tracheostomy decannulation was performed. During his hospitalization, pulmonary tuberculosis was diagnosed, and the patient received anti-tuberculosis medications. The patient had smoked approximately two packs a day for 40 years prior to being infected with COVID-19 but quit smoking afterward. Prior to this, the patient had no issues with his daily life. On 27 September, the patient was transferred to our hospital’s Department of Physical Medicine and Rehabilitation for comprehensive rehabilitation treatment, including PR. At the time of admission, a manual muscle test on his upper extremities showed a Fair grade, while the proximal part of his lower extremities was graded as F, with particular weakness in the quadriceps muscle, and the distal part graded as G. His static sitting balance was well maintained, but his dynamic sitting balance was poor. Moderate assistance was required for chair-to-stand movement, and independent standing was only possible for a few seconds. With the use of a walker, supervised walking was possible for distances of less than 100 m, but walking without a gait aid was not possible. Moderate assistance was required for activities of daily living. Chest X-ray revealed diffuse ground-glass opacity with a large nodular lesion in the left upper lobe (Figure 1). Most of this patient’s measurements in pulmonary function, balance, and exercise capacity were outside the reference values or the expected values for a healthy individual (Table 1) [15,16,17,18,19,20]. The standard 30 min PR session included deep breathing exercises, inspiratory muscular training, ergometer exercises, and walker gait training, with all being supervised by a therapist. The RAGT intervention was conducted from October 12 to November 17, with a total of 22 treatment sessions administered. RAGT was initiated after his standing balance was sufficiently established and supervised walker ambulation was possible at minimal distances in order to facilitate effective gait training. Morning Walk^®^ (Curexo, Seoul, Republic of Korea), an end-effector type robot, was applied for the RAGT. This robot device is composed of a saddle seat and two handlebars that measure body weight support (BWS), a footplate that reproduces the stance and swing phases in the lower extremities during gait, and a monitor that provides visual feedback on movement (Figure 2) [14]. We applied the ground mode in RAGT to improve the subject’s functional mobility, with the following gait parameters set: a cadence of 30 steps per minute, a stride length of 38 cm, a step height of 6 cm, an initial contact angle of 5 degrees, a toe-off angle of 30 degrees, and BWS of 20% bilaterally. During the gait task, if the subject maintained a Borg rating of a perceived exertion grade ≤ 10 or a ground reaction force of 50% or higher, we increased the cadence value by 5 steps/min (0.1 km/h) within each session [14]. To progressively increase the difficulty level between the RAGT sessions, we set the terminal cadence value achieved in the prior session as the initial cadence value for the next session. Additionally, we used the mean BWS value calculated in the previous session as the setting value for the subsequent session. As a result, we recorded the initial and final cadence values, along with the mean BWS, for each session to evaluate the patient’s task-specific ability and voluntary postural control within the program. However, if the patient encountered difficulty during a session, the cadence and mean BWS values were reverted back to those used in the previous session. After all treatment sessions, there was an improvement in both his MIP and MEP in terms of pulmonary function, with these values increasing from 52 and 59 cmH20 to 58 and 69 cmH20, respectively, along with an increase in FVC. Additionally, the patient’s lower extremity muscle strength mostly improved (Table 1). In terms of balance ability, the patient demonstrated improvement, with his scores increasing from 4 to 10 points in the SPPB. The time taken during the TUG improved from 29.37 to 13.14 s. In terms of his exercise capacity, the patient’s 6MWT distance improved from 66 to 240 m, while he also showed an improvement in oxygen saturation. Furthermore, his FAC scores improved from 2 to 4 points. His COVID-19-related symptoms, including as assessed by the BFI, CAT, and LCADL, exhibited improvement (Table 1). Furthermore, in the first session of the RAGT program, the initial and terminal cadence values were 30 and 30, respectively. While these parameters gradually improved over subsequent sessions, challenges arose during the 15th treatment session, marked by symptoms such as shortness of breath and fatigue in the patient. Consequently, the session was maintained at the same intensity as the preceding one (14th), with the initial and terminal parameters set at 45 and 50, respectively. This level persisted throughout the remaining treatment sessions. Additionally, there was an overall improvement in the average BWS per session, with the amount of BWS reducing from 15% in the first session to 3% in the last session (Figure 3). Due to the retrospective design, the requirement for informed consent was exempted by the National Health Insurance Service Ilsan Hospital Institutional Review Board. This report was provided with informed consent from the individual. From 5 June 2023 onward, all data pertaining to the patient were obtained for research purposes.

## 3. Discussion

This report demonstrates the impact of using RAGT as a tailored rehabilitation approach for ambulation dysfunction following COVID-19. Currently, there is a lack of extensive research on the application of RAGT specifically to patients with lung diseases, including COVID-19, as well as the establishment of tailored rehabilitation programs for post-COVID-19 individuals with impaired functional ambulation [14,21,22]. Our results showed that applying the PR program alongside RAGT improved the pulmonary function, lower extremity muscle strength, balance, exercise capacity, functional ambulation, and COVID-19-related symptoms in a patient with ambulation restriction due to severe COVID-19 pneumonia.

In terms of pulmonary function, previous studies have demonstrated that RAGT can improve the respiratory ability in individuals with neurological disorders with poor mobility [23,24]. Additionally, brisk and vigorous walking training has been shown to improve cardiopulmonary function in patients with cardiopulmonary diseases [25]. RAGT can modulate the difficulty of aerobic fitness for individuals with mobility impairments by utilizing various parameters, such as walking speed and BWS. This approach allows for the adjustment of the difficulty of walking while maintaining safe mobility, enabling patients to achieve the intensity threshold required for meaningful improvements in their cardiorespiratory function [10,26]. In this report, the patient adhered to a progressive difficulty protocol during RAGT, successfully achieving the goals of an improved walking speed and a reduced BWS.

In terms of lower extremity strength and balance, improvements were observed. Many studies have reported that robotic-assisted training increased the lower extremity muscle activity in patients with neurological disorders and elderly individuals [12,13,27,28]. In a study conducted on older adults with cardiovascular disease, it was found that robotic-assisted training improved their balance ability [11]. Additionally, the application of RAGT could enhance the balance ability of patients with neurological disorders, as well as that of outpatients following severe COVID-19 [14,27,29]. The patient had significant impairments in his balance ability and lower extremity motor strength, which were caused by the prolonged absence of mobilization of the lower extremities due to severe COVID-19 pneumonia. Therefore, the application of RAGT would have specifically involved voluntary muscle activation and facilitated voluntary postural control, assisting the subject in achieving a high level of ambulation, such as standing with robot-assisted support. As a result, these improvements in muscle strength and balance are likely to have contributed to an improvement in his functional ambulation level. This is consistent with many findings in subjects with neurological disorders and functional ambulation restrictions that RAGT results in improvements in the FAC [30,31].

In the assessment of his exercise capacity, improvements were observed, as were improvements in exercise-induced desaturation, as indicated by SpO2%. Using RAGT may help ensure that COVID-19 patients with reduced mobility experience functional mobility earlier. Combining RAGT with a PR program can further improve exercise capacity, as evidenced by previous studies that have shown an improved exercise capacity in COVID-19 patients through using a combination of functional movement exercises and respiratory therapy [32,33,34,35]. A previous report also showed that a combination of RAGT and respiratory training led to improvements in exercise capacity, with desaturation improved in patients following severe COVID-19 [14].

Furthermore, his COVID-19-related symptoms improved after treatment. This aligns with the conclusions of various studies on COVID-19 rehabilitation [8,33,36]. Furthermore, meaningful improvements were observed in the SPPB, BBS, TUG, and 6WMT with an MCID score ≥ 1, an MDC score ≥ 5.9, an MDC ≥ 2.68 s, and an MCID ≥ 30.5 m, respectively [37,38,39,40]. The patient exhibited significant improvements in his task-specific abilities during the RAGT program, as reflected in enhancements in both his initial and terminal cadence values, as well as the BWS in the last session (Figure 3). These improvements signify a positive shift in task adaptability and enhanced voluntary postural control, respectively.

Namely, we contemplate that these improvements may enhance the feasibility of applying PR alongside RAGT for COVID-19 patients with functional ambulation limitations.

This report had several limitations. First, it did not include a control group, and the sample size was restricted to only one individual, as it was a case study. Second, the absence of a follow-up test prevented the evaluation of the retention effect of the intervention. Third, there is a lack of comparable studies, such as randomized controlled trials, to support the findings of this report with specific cases. However, this report is particularly notable, as previous studies have primarily focused on rehabilitation programs for individuals residing in the community or outpatient settings, as well as those who are ambulatory during inpatient rehabilitation [6,8,33,35]. Fourth, due to the design of the case report, there are limitations in assessing the effects of pure RAGT compared to other PR training methods on this subject. Considering the aforementioned limitations, future research should conduct comparative analyses using a variety of interventions (e.g., PR vs. PR + RAGT vs. RAGT) and diverse participant groups, including patients with both ambulatory and non-ambulatory capabilities. Our report identified improvements in pulmonary function, lower extremity muscle strength, balance, exercise capacity, and functional ambulation using PR accompanied by RAGT, showcasing effective adaptation to the RAGT program. This report provides valuable insights for developing efficient rehabilitation programs tailored to inpatients with functional ambulation restrictions resulting from severe COVID-19 pneumonia. These findings suggest enhanced potential for realizing the benefits of integrating RAGT into PR for this specific patient population.

## Figures and Tables

**Table 1:** Comparison between before and after treatment.

	Before Treatment	After Treatment	Reference Value
**Exercise capacity**			
6MWT, m	66 ^a^	240 ^a^	>572 ± 92 ^b^
Pre/post SpO2, %	98/88 ^a^	99/98 ^a^	
1STS, count	0	15	33–48 ^c^
Pre/post SpO2%	99/90	98/96	
**Physical function**			
SPPB, score	4	10	>9.8 ± 1.7 ^b^
BBS, score	29	48	>55 ± 1 ^b^
TUG, sec	23.97 ^a^	13.14	<8 ± 2 ^b^
FAC, score	2	4	
**Pulmonary function**			
FVC, mL (predicted, %)	1880 (43)	2280 (52)	
MIP, cmH20 (predicted, %)	52 (55)	58 (61)	
MEP, cmH20 (predicted, %)	59 (47)	69 (55)	
**Lower extremity muscle strength**			
Right hip flexor/left flexor, N Supine with hip flexed at 90 degrees	5.7/7.3	7.1/8.6	
Right. hip flexor/left flexor, N Supine with hip flexed at 45 degrees	6.4/5.0	7.4/7.1	
Right knee extensor/left extensor, N Sitting	4.8/6.2	5.9/6.4	
Right knee extensor/left extensor, N Supine	4.5/5.3	6.7/5.9	
Right ankle dorsiflexor/left flexor, N Supine with knee extended	6.0/3.8	4.7/7.5	
Right ankle dorsiflexor/left flexor, N Supine with knee flexed	4.3/3.0	7.3/8.4	
Right ankle dorsiflexor/left flexor, N Supine with knee extended	5.1/7.7	9.9/8.2	
Right ankle plantar flexor/left flexor, N Supine with knee flexed	7.6/7.4	13.5/9.8	
**PROM**			
BFI, score	4.4	3.4	
LCADL, score	56	38	
CAT, score	30	21	
FES-I, score	50	48	<23
**Quality of life**			
Physical domain, score	50	38	73.5 ± 18.1 ^b^
Psychological domain, score	50	50	70.6 ± 14.0 ^b^
Social domain, score	44	44	71.5 ± 18.2 ^b^
Environmental domain, score	44	44	75.1 ±13.0 ^b^

^a^, with an anterior walker; ^b^, mean ± standard deviation; ^c^, median in interquartile range (25–75th percentile). MIP, maximal inspiratory pressure; MEP, maximal expiratory pressure; SPPB, short physical performance battery; BBS, Berg Balance Scale; TUG, timed up and go test; FAC, Functional Ambulation Classification; 6MWT, 6 min walk test; 1STS, 1 min sit-to-stand test; PROM, Patient Reported Outcome Measures; BFI, Brief Fatigue Inventory; LCADL, London Chest Activity of Daily Living; CAT, COPD Assessment Test; FES-I, Falls Efficacy Scale-International.

## Data Availability

Data sharing is not applicable to this article, as no datasets were generated or analyzed during the current study due to its design as a case report.
